# Tuberculosis vaccines: Rising opportunities

**DOI:** 10.1371/journal.pmed.1002791

**Published:** 2019-04-23

**Authors:** Johan Vekemans, Katherine L. O’Brien, Jeremy Farrar

**Affiliations:** 1 World Health Organization, Initiative for Vaccine Research, Geneva, Switzerland; 2 Wellcome, London, United Kingdom

## Abstract

Johan Vekemans, Katherine O'Brien, and Jeremy Farrar discuss recent breakthroughs in the search for a highly effective tuberculosis vaccine.

A vaccine preventing pulmonary tuberculosis (TB) in adults is needed but has long been considered an elusive goal. Times are changing in the field of TB vaccines, though, with recent results boosting confidence in the feasibility of a TB vaccine with potential as a decisive tool in the fight against TB.

*Mycobacterium tuberculosis* (Mtb), the causative agent of TB, is the leading cause of death from any single infectious pathogen. In 2017, an estimated 10 million people developed TB, and 1.6 million people died of the disease. Approximately 1.7 billion people—23% of the world’s population—have latent TB infection (LTBI) and carry the risk of developing TB during their lifetime. The emergence of Mtb strains resistant to TB drugs causes a major growing burden of hard-to-treat infections. An estimated 558,000 people developed drug-resistant TB in 2017, 82% of which were multidrug-resistant cases; 230,000 deaths were due to drug-resistant TB [1].

Important efforts are being directed to TB control through the WHO End TB Strategy, which set ambitious targets for reduction of disease burden. However, current trends fall well short of those needed to meet the goals [1]. Last year, the United Nation’s High-Level Meeting on TB renewed the commitment to fight TB, but it is clear that current approaches are insufficient, highlighting the importance of research and development for new tools [2]. Disease impact and health–economic modelling has shown the value of a vaccine that would prevent pulmonary TB in adults not only for those immunized but also by reducing transmission to others [3].

Such a vaccine, long considered an elusive goal, may now be close at hand, given new clinical trial results from a Phase 2b trial in South Africa and Zambia [4]. Two doses of the M72/AS01E, an adjuvanted fusion protein construct based on two TB antigens, was shown to provide 54% (90% CI 14%–75%) protection against pulmonary TB in individuals with LTBI over an average 2.3 years of follow-up. Secondary analyses, based on a limited number of cases and therefore subject to caution, suggest that there was no waning of effect over time and that protection may be highest in younger individuals. Data from follow-up through an additional year are awaited in the coming months. In this study, the point estimate of vaccine efficacy was above what had been predefined as a preferred lower target level by WHO [5].

The lack of reliable models to predict human protection in early clinical development did not allow confidence building in this product before the trial results were known, and further investments had not been planned. Decisions are now needed for financial investments to support further clinical development, progression to Phase 3 evaluation, and preparation of the downstream pathway to affordable access and use.

Various clinical development options should be considered. A vaccine with characteristics as demonstrated in the Phase 2b trial may be of significant interest in endemic regions characterized by high attack rates, where most young adults have been infected. Proof-of-concept remains to be established for people who don’t have LTBI, especially for geographical regions where transmission intensity is lower. As existing results suggest the vaccine prevented the natural course of progression from infection to pulmonary disease, it would be relevant to investigate similar immunotherapeutic effects in subjects who live or lived in contact with TB patients or in subjects known to have recently converted diagnostic markers of infection. Testing for use as a therapeutic adjunct to improve outcomes of drug treatment in TB patients should also be considered. Children, older individuals in countries where the epidemic is driven by TB reactivation [6] and specific high-risk groups such as HIV-infected people, should also be considered for evaluation in order to not be denied a potential protective intervention.

Although advancing the evaluation of M72/AS01 is now a major priority, it is not the only important progress in the TB vaccine field.

Another promising breakthrough emerged from a recent study in South Africa evaluating the effect of Bacillus Calmette-Guérin (BCG) revaccination in people vaccinated with BCG at birth and with no evidence of LTBI [7]. The coprimary endpoints of this trial were not achieved; however, secondary analyses suggested that BCG revaccination reduced the proportion of sustained conversion of in vitro markers of LTBI by 45%. The clinical significance of this new finding is unknown, especially in light of past studies that have shown no impact of BCG revaccination on TB [8–9], which formed the evidence base for WHO not recommending BCG revaccination [10]. The risk of disseminated BCG disease in subjects with immunosuppression would constitute an important obstacle to BCG revaccination strategies in HIV and TB coendemic areas. This research signal nevertheless constitutes an important opportunity to characterize immunological mechanisms of protection against Mtb infection, and such investigations are planned.

Recent early-stage developments are also cause for optimism. As presented in a recent review, new constructs in preclinical testing include recombinant cytomegalovirus (CMV)-based candidates inducing atypical immune responses and supporting investigations in previously unexplored territory in the science of TB vaccinology; new imaging techniques allow for monitoring of TB progression in vaccinated primate models of experimental infection; attempts to develop safe human models of experimental infections are being developed; the role of the route of vaccination is being explored; and immune markers of TB risk are increasingly being identified, with the potential to inform rationale vaccine design, testing pathways, and eventually support regulatory-acceptable accelerating bridging steps [11]. The pipeline of other products in clinical development is diverse, with a variety of live-attenuated or killed mycobacteria-derived candidates (DAR-901, MTBVAC, RUTI, Vaccae, VPM001), adjuvanted recombinant proteins (H56:IC31, ID93/GLA-SE), and recombinant viral vectors (MVA85A, ChAdOx1.85A, MVA85A, Ad5 Ag85A, MVA85A-IMX313, TB/FLU-04L), progressing through human evaluation [12].

While the statements from the UN High-Level Meeting are welcome, transforming discovery and vaccine candidates into products that can have impact takes more than declarations of support. Funding levels, unfortunately, are insufficient, and the US$1.3 billion annual funding gap in TB research needs to be filled [12]. More funding should be targeted to support TB vaccine research, which has been estimated at US$74 million in 2017, a surprisingly low figure as compared to the US$174 and US$700 million allocated to, respectively, malaria and HIV vaccine R&D [13] when considering the scientific opportunity, unmet need, and investment case [14].

Progress toward TB elimination will require vigorously pursuing the potential contribution of novel TB vaccines propelled by new evidence recently made available. Advocates working on TB as a major global health problem should lead decision-makers toward this realization. The possibility to transform the opportunities into action will be a test of the collective ability of the global health community to succeed in developing and using vaccines aimed at addressing diseases disproportionately affecting the poor. The world needs better operating models supporting prompt progress from vaccine efficacy proof-of-concept to evaluation for use and impact in support of policy decision and funding for implementation. A sense of responsibility toward global health from the corporate sector manufacturing vaccines is needed as well as from the public and philanthropic sector, through the setup of functional public–private partnerships supported by innovative funding mechanisms. Opportunities are rising in the search for tools to prevent TB; let us not squander this chance. Now is the time for mobilization toward vaccine impact against TB.

## Abbreviations

BCG: Bacillus Calmette-Guérin
CMV: cytomegalovirus
LTBI: latent TB infection
Mtb: *Mycobacterium tuberculosis*
TB: tuberculosis
